# DISNET: a Distributed Instrument System NETwork

**DOI:** 10.1155/S1463924684000067

**Published:** 1984

**Authors:** Paul J. Gemperline, Robert Megargle, Arthur Dartt, Larry Slivon, Victor Zadnik

**Affiliations:** 1 Department of Chemistry Cleveland State University Euclid Avenue at 24th Street Cleveland Ohio 44115 USA; 2 Department of Chemistry East Carolina University Greenville North Carolina 27834 USA


Journal of Automatic Chemistry, Volume 6, Number 1 (January–March 1984), pages 27–32

### DISNET: a Distributed Instrument System NETwork

**Paul J. Gemperline,* Robert Megargle,† Arthur Dartt, Larry Slivon and Victor Zadnik**
*Department of Chemistry, Cleveland State University, Euclid Avenue at 24th Street, Cleveland, Ohio 44115, USA*

The ability to share resources among computerized instruments is a major innovation offered by computer networks [1]. The resources may consist of any combination of (*a*) information, (*b*) computing power, and (*c*) peripheral devices. The ultimate purpose of such a network is to provide better computer services at the point of need in the most cost-effective manner. Current work in the field of computer and information science is addressing these concepts; a book, *Computer Networks*, provides an excellent introduction and review of this work [2].

Several other advantages are offered. First, a properly designed laboratory network system will be expandable, allowing new instruments, computers, and additional capability to be easily added. Greater reliability can be offered. Isolated computer failures in the network can be replaced with alternate sources of service. Individual stations are easier to design, with each station programmed to provide a specialized service rather than using one highly sophisticated unit to provide for every possible need. Development of new instrumentation and techniques is facilitated. Powerful hardware and software may already be available in the network to support the development of new technology. Finally, expensive computer peripherals can be efficiently used by many computerized instruments. Concise data can be sent over inexpensive communication media for storage or output.

### Desired network properties

There is some disagreement in the literature as to what constitutes a computer network [2]. A computer network is defined here as the 'hardware and software components of a communication system which interconnects autonomous computers'. The definition is meant to exclude 'time-sharing' and 'multiprocessing' computer systems.

Local area networks may be implemented with numerous point-to-point connections between computers. They require appropriate software to route messages through the various data pathways. A different approach is to connect the computers to a common data pathway that is shared in time by all users. Computers communicate by broadcasting messages directly to all other computers. Only a subset of all the computers may choose to listen to the message, depending on address information included in its heading. A system consisting of serial transmission over coaxial cable is common.

In a laboratory, common data pathway networks have certain advantages. Any computer can communicate directly with any other. The need for buffer space and computer power to receive and then forward messages to other destinations (like packet switching or other point-to-point networks) is eliminated. Each node can be more easily implemented with the kind of computers normally found with laboratory instruments. Of all the network topologies that have evolved, only the bus, star, and ring are suitable for the broadcast technique (see figure 1). Bus structures can include branching and are most widely used for broadcast networks.

It is also desirable that a network should not require a central controller since its failure would disable the entire system. Some control mechanism is needed, however. The star requires a central control system and is therefore less desirable. Many ring and bus topologies are also designed with centralized control. This is not a requirement, however, and they can be implemented with the advantage of decentralized control.

Lastly, some networks employ a token passing scheme in which use of the common broadcast media is allocated in turn to each node. This scheme is easily implemented for rings and stars, but is complex in bus designs. Token passing is desirable in applications where most nodes need to use the network most of the time. In a laboratory, however, the network is used infrequently by various instruments and laboratory stations, and a random access mechanism is better.

### *Control structures*

In broadcast technologies, a collision occurs when two or more stations transmit simultaneously. A mechanism is required to either prevent or to detect and correct these collisions. Packet integrity may be verified by the receiver through check words within each packet. Collisions are assumed to fail the check. The receiver sends an acknowledgement after some number of properly received packets. A collision is assumed to have occurred and the packet is retransmitted when no acknowledgment is received [3].

Several busy detection schemes have been developed to increase network throughput by decreasing collisions and station wait time. In one example, the sender is required to 'listen' before sending so that it will not interrupt transmissions already in progress [1]. The popular ETHERNET system uses this scheme, and in addition 'listens' to itself while it sends to detect collisions resulting from simultaneous transmission by another station [4]. In this case, both stations stop early and try again later after a random time interval.

### DISNET

This paper describes a Distributed Instrument System NETwork (DISNET) which was implemented within the Department of Chemistry at Cleveland State University. Its purpose is to support laboratory data acquisition, instrument control, and experimental data processing. DISNET is a linear bus computer network using the broadcast concept and employing random access control. It allows any two stations to communicate and requires no external computer or master to control the activity. The present line is over 750 ft in length and the maximum is probably about 2000 ft. The transmission media consists of 15

* Present address: Department of Chemistry, East Carolina University, Greenville, North Carolina 27834, USA.
† Author to whom correspondence should be addressed.

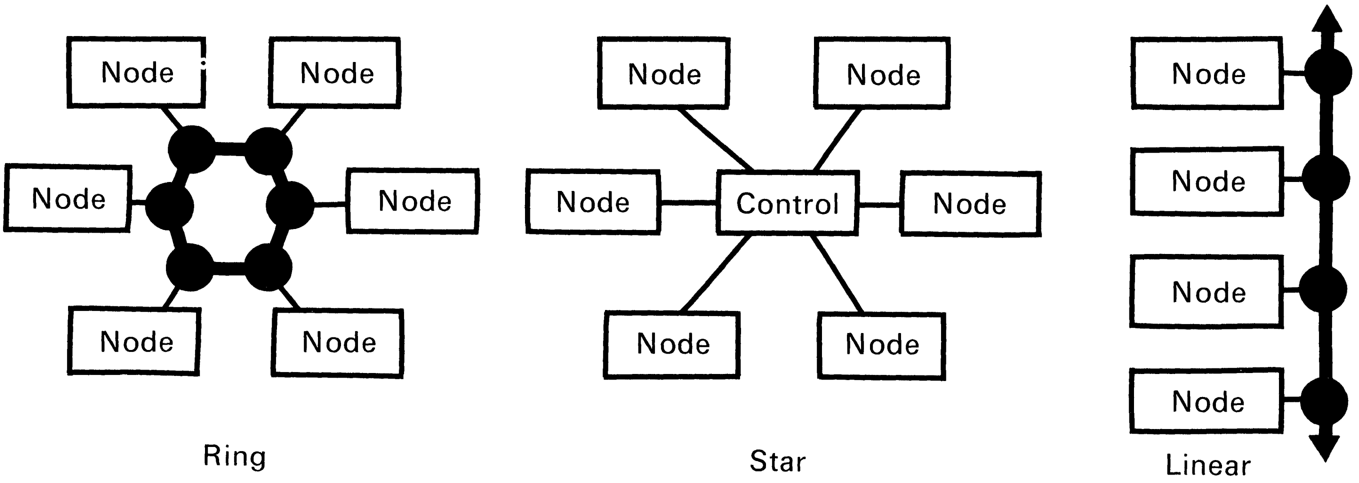

*Figure 1. Network configurations suitable for broadcast-type networks.*

twisted pairs of wire; eight pairs for data, three pairs for status and four pairs for control. Each twisted pair is individually shielded and driven at 6 mA by differential line drivers (type 75109) for high speed and good noise immunity. Matching line receivers (type 75107) are used to receive the data.

The hardware was designed to provide automatic address recognition and sufficient status and control information to accommodate 'dumb' stations. The addressed partner is called the slave. Hardware arbitration is provided to determine which station is allowed to gain control when there are simultaneous requests to control the line. The hardware also provides time-out mechanisms that will prevent controlling stations from halting all activity on the network when malfunctions occur.

The transmission hardware can support data transfer rates of up to 1 M bytes/s and should rarely limit the communication speed between computers or instruments. The hardware also provides for asynchronous operation. This allows dissimilar computers to communicate with each other. When fast computers communicate with slow computers, the slower partner in the transaction will limit the data transfer rate.

The cable for the network has been installed in the fourth floor of the Cleveland State University Science Building where the Department of Chemistry resides. Figure 2 gives the floor plan of the fourth floor; the wide black line shows the path of the network. Circles represent drop points where stations can be tied into the network—these points are actually boxes mounted on the walls with cable connectors installed to accommodate the send/receive hardware.

The DISNET hardware was conceived in 1973; by 1975 design work had been completed, limited funding obtained, and construction was underway. Prototype interfaces were soon constructed and the data transmission hardware was tested under computer control. In 1979, development of interface hardware and software was started to implement the first application of the DISNET system, and this system became operational in 1981. During these years, the onslaught of inexpensive microcomputers greatly changed the face of computer networking. The system thus evolved during a time when the field of computer networking itself was in a state of evolution.

### Hardware description

An overview of the basic send/receive hardware is given in figure 3. The circuit is constructed on one printed circuit card and controls the line acquisition sequence, the address transmission and recognition sequence, the data transmission sequence, and the receive sequence. Connection to the transmission line is made through a 36-pin single row edge connector (transmission line wires are represented as wide dark lines in figure 3). These include three pulse code lines, eight data lines and the control signals called ANSBK and SNDREC. All 13 are bi-directional. The line drivers in each station are normally disabled so the bus can be used in a party line fashion by many users. The two other control signals are called BUSY and PRIORITY—they are daisy chains propagating in opposite directions.

Control and data signals are provided for interfacing instruments or computers through a 22-pin double row edge connector (they are shown as narrow lines in figure 3). Since the system was designed to allow interfacing to 'dumb' nodes as well as 'intelligent' computer nodes, the input and output pulse code and data lines are separate. A pulse applied to an input pulse code line causes data to be transferred and a pulse to occur on a corresponding pulse code output line of the other station. These output pulses may be used as strobes to route data to up to six different hardware registers at the receiver.

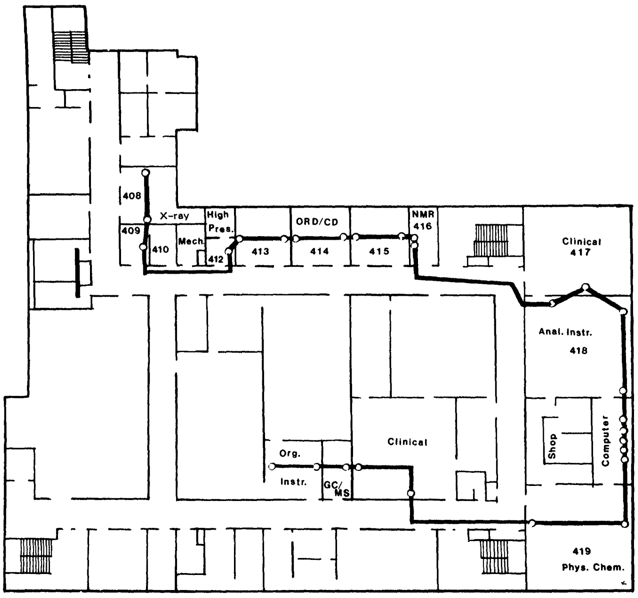

*Figure 2. Route of DISNET through the Chemistry Laboratories of Cleveland State University.*

### *The acquisition sequence*

Network acquisition is implemented with the BUSY and PRIORITY lines. Both lines are received and then retransmitted

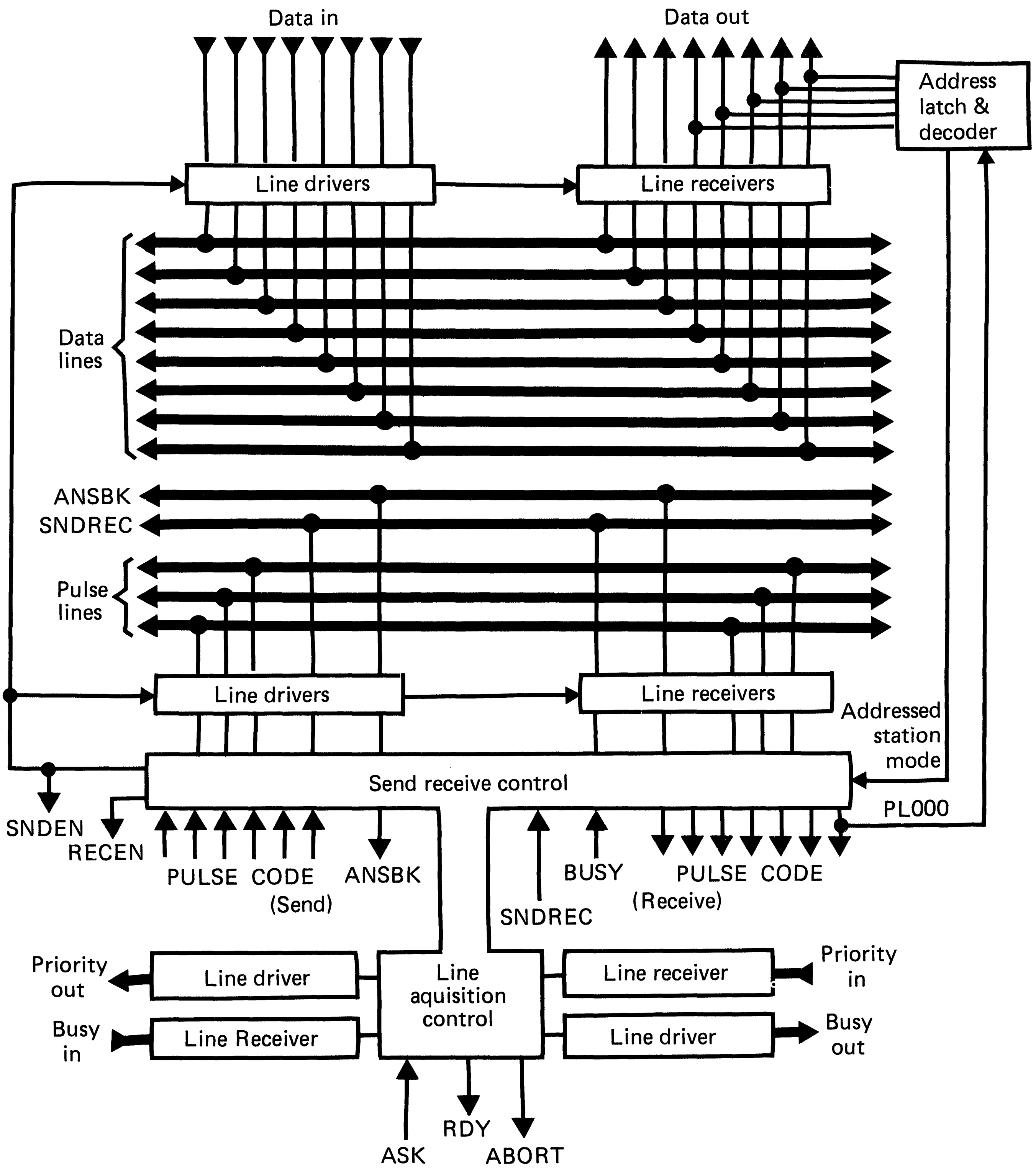

*Figure 3. Functional organization of a DISNET station.*

at the same or modified logic level to the next station, but in opposite directions. Unused station connectors must have a jumper card installed to preserve the continuity of these signals. Stations normally retransmit the same signal they receive unless they are controlling or attempting to control the transmission line. In that case, the station transmits a zero out both lines. A station is prevented from attempting to control if it receives a zero at either the BUSY or PRIORITY input line. The priority of a station is determined by its location in the line. For a station X in the middle of the line, all stations in the direction which receive the BUSY signal are higher priority and all stations in the other direction are lower priority.

Figure 4 is a schematic diagram of the basic Send/Receive circuit. External circuits at station X can initiate the acquisition sequence at any time by bringing the ASK- line low. If the busy input line BIN-is high (indicating that no lower-priority stations are controlling the line) then a low is sent to the input of the request flip-flop FFI. If the PRIORITY line is also high (indicating that no higher-priority stations are controlling), then flip-flop 1 sets and R- goes low. This sends zeros out on the PRIORITY and BUSY lines to inhibit other users and starts the DELAY monostable. This time delay is adjusted to ensure that the BUSY signal has time to propagate to the high-priority end of the line. Any higher-priority station Y may overide the lower-

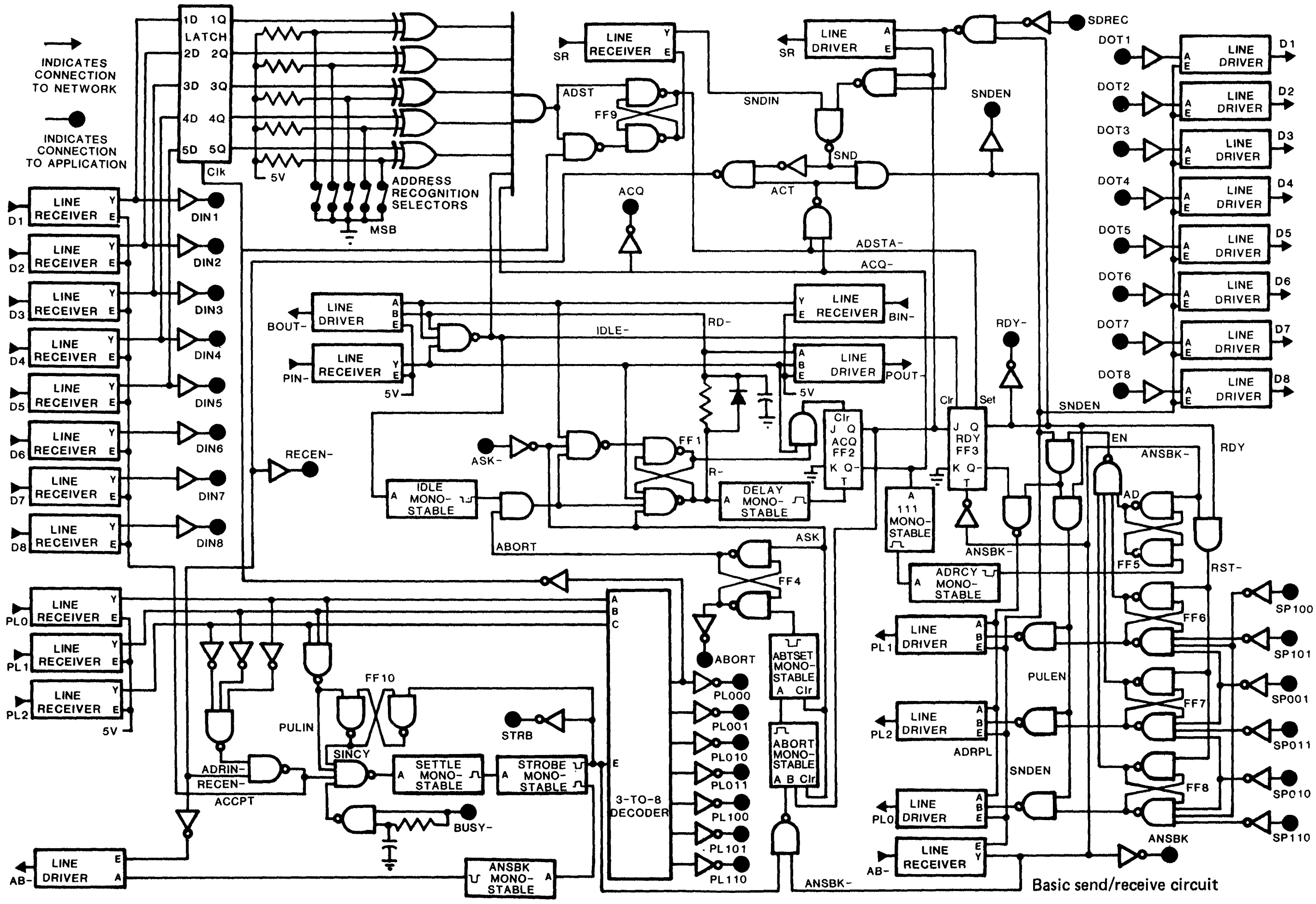

*Figure 4. Logic diagram of the DISNET send/receive circuit.*

priority station X during this time delay by sending a zero to X on the PRIORITY line. This process resets station X's request flip-flop, FFI, to defer its acquisition request. Station X's request will automatically be processed again when the network is available. During the delay time, the circuit asking for the network ignores any BUSY signals it may see. Lower-priority stations that are also asking will defer when they receive the low on BUSY coming from this station. At the end of the delay pulse, all other stations are guaranteed disabled and the trailing edge of the pulse sets the ACQ flip-flop, FF2. This signals that the network is ready for data transmission.

### *Address transfer*

The pulse code lines are normally left in the 111 level. They are biased slightly high so this is the normal level when the transmission line is idle. All stations are always enabled to receive pulse code information. When these lines are changed to some code other than 111, a transmission of data is initiated. A pulse code of 000 designates the transfer of a station address. Station addresses use only the least significant five data lines in this implementation, giving a maximum of 32 stations. Expansion to 8 bit addresses and 256 stations is trivial.

When the ACQ flip-flop sets in a station that is controlling the network, ACQ- goes low and starts the 111 monostable. This in turn pulses the ADRCY monostable to set FF5, the address transfer flip-flop. The 111 monostable ensures the pulse code line drivers transmit 111 for a time after they are enabled. When FF5 sets, signal AD goes low and EN goes high to allow a transmit cycle. Since the RDY flip-flop is clear, ADRPL will go low during the send time to cause all pulse lines to be set to zero. This is the code for the address transfer. It is important that the correct destination address data be placed on the data lines by the external application during this send cycle.

### *Address recognition*

All other stations receive the 000 pulse code which causes data to be latched into the address register. A series of switches are wired so that only one code results in a high signal at ADST. Each station has a unique code or address. When PL000 goes high in the station whose address code matches the sent address, ADSTA goes high and the RDY flip-flop sets—indicating the station is addressed as a slave. The RDY flip-flop will not set in any other station.

### *The send sequence*

The SDREC line is always set by the station that has control of the transmission line. This line determines whether the controlling station or the addressed station will send data. The other always receives. The send sequence is possible only if the station is in the send mode as indicated by a high on the SNDEN line. This signal enables the pulse code line drivers, the data line drivers, and the answer-back receiver.

The EN signal is brought high to enable a send sequence when one of the pulse code inputs (001 to 110) is given a high pulse by an external circuit. Such a pulse clears some combination of the pulse code flip-flops, FF6 to FF8. This begins a period of time called the 'send time'. Correct data should exist on the data line inputs throughout this time. The pulse code flip-flops serve to latch the pulse code. The inverted output of the flip-flops are gated to the pulse code line drivers when PULEN goes high during the send time. The line drivers have gated inputs and can be wired in such a way that a low will be transmitted if either input A or B is low. The drivers will only attempt to drive the line if the enable input, E, is high.

The send time is terminated by the ANSBK- pulse from the receiving station. Flip-flops FF5 to FF8 are reset to their inactive states by the ANSBK- and RST- signals to end an address or send cycle. The flip-flops causing the send are reset as soon as the low edge of the ANSBK- pulse is received. This low signal is transmitted by the receiving station for sufficient time to ensure the pulse code lines return to 111. In that way the next transfer pulse code will be recognized. The RDY flip-flop in the controlling station is also set by the first ANSBK pulse obtained from ANSBK-. It is important that the high signal from the external circuit on one of the pulse code input lines (which originally caused the pulse code flip-flops to clear) be returned to a low before the RST- pulse is over. Also, a second transfer with a new pulse code should not be started until the ANSBK- pulse is over. The external circuit may use ANSBK- to initiate the process of sending the next piece of information. The send time may be abnormally terminated if the controlling station goes off the line for some reason, causing IDLE- to go low.

A slave station may also send. At slave stations, the ACQ flip-flop is not set and the SNDEN signal is controlled by the SNDREC line receiver using data sent by the controlling station. Except for these differences, the send sequence at a slave is the same as at a control.

### *The receive sequence*

Figure 4 shows that the pulse code receivers are always enabled. The receiver can sequence if BUSY is low and either RECEN- is low or pulse code 000 is received. RECEN- will be low if this station is an addressed or controlling station and is in the receive mode. BUSY is controlled by the external circuits and is provided for external devices that may not be finished processing the previously received byte. If the station holds the BUSY input high, the receive circuit operation is simply delayed until the high input is removed or until the ABORT monostable ends the operation. A low signal at RECEN- makes ACCPT high to enable the data line receivers.

The SETTLE monostable is started whenever the pulse code changes from 111. This monostable provides a slight time delay to allow the information to stabilize on the lines before attempting to use it. Among other advantages, it allows for slight differences in transmission time on different lines. A STROBE pulse is generated at the end of the SETTLE time which causes the 3-to-8 decoder to set one of its outputs low depending on the ABC inputs. The output lines, PL000 to PL110 can be used by external circuits to route data to the correct register. In addition, the strobe pulse sets SINCY low so that PULIN must go low (pulse lines set to 111) before another cycle can be started.

The strobe pulse starts the ANSBK monostable. The ANSBK line driver is enabled by RECEN to return the ANSBK pulse to the sending station. Receipt of that pulse is proof to the sending station that the receiving station accepted the previous data byte.

### *Abort sequence*

Because the transmission line can accommodate many users and errors can occur, a protection mechanism is included. A retriggerable ABORT monostable is started whenever a station gets acquisition and causes the ABORT monostable's B input to go high. The pulse length is set for the longest time needed to complete a transfer of one byte. If the station has not completed its transfer by the end of the ABORT pulse, an ABTSET monostable is pulsed to set the ABORT flip-flop FF4 and force the station off the network. Pulses on the STRB- and ANSBK- lines indicate that transfers are occurring. Each retriggers the ABORT monostable so that no abort action is taken if line activity is occurring at a reasonable rate. The ABORT flip-flop is reset when ASK- is returned high.

### *Termination sequence*

When a controlling station is finished using the transmission line, it allows the ASK- line to go high. This clears the request flip-flop which in turn clears the ACQ flip-flop. Whenever the transmission line is in use, the BIN- or PIN- signal in every station except the controlling station is low. In the controlling station RD- is low. The IDLE monostable in every station therefore pulses when the controlling station gives up control. This enforces an additional delay before another station can attempt to gain control of the network; the delay ensures that the control and slave stations previously in use will be reset by the low on the IDLE- line. The small time delay between R- and RD- when R- goes low is included to ensure the IDLE monostable has time to trigger when any other station gives up control and station X has a request pending. The IDLE monostable also provides an opportunity for the system manager to adjust the priority importance of stations, independent of their position in the transmission line. The longer the IDLE pulse, the more opportunity there is for important stations to gain control of the network.

When a station has control of the network and gives it up by letting ASK- to go high, it can then immediately request the line again to address a different station. ASK also clears the ABORT and ABTSET monostables so that a closely following second request is not hung up by an ABORT pulse just finishing from the previous use.

### Implementation and testing

Two DISNET stations were constructed first and put into use. Prototype interfaces were built and initial test programs written for a Texas Instruments 960A minicomputer and an ALTAIR 8080 S-100 microcomputer system. The prototype interface provides a 16-bit parallel input/output path for data and control and was used for both computers. The test programs were designed to gain control of the network, alternately send or receive a 64-byte data record, drop acquisition of the network, check the received data, and print a dump when errors were detected. A time delay was performed between the transmission of each record to ensure that the communicating computers remained synchronized.

The test was allowed to run for about 1330 h, during which only one record was found to be in error. Transmission of the record in error coincided exactly with the occurrence of a lightning bolt which struck a nearby telephone pole. The test programs reported the error and continued properly with the next transaction. No further errors were detected and the test program was finally shut down after 4·22 M records of data were successfully transmitted, corresponding to over 270·5 M bytes of data. The system has since proven to be equally reliable.

### Comparison with other networks

DISNET offers some advantages for its intended purpose over the broadband and baseband networks that are now becoming popular. The pulse codes that allow information to be routed to particular registers at a receiver, and the ability of a computer to call another site and request data from it, are unique. These features allow a computer to collect data from, and provide simple control of a non-computerized instrument or experiment at a remote site. The pulse codes can also be used to identify the type of data byte being sent: data, control code, error code, and check character. This, plus the line reversal feature, allows computer-to-computer communication to incorporate error checking and acknowledgement within each packet. There is no need for separate acknowledgements. Since the acquisition scheme does not permit collisions, retransmission of damaged data is rarely needed. The last two features result in more efficient use of the communication medium.

On the other hand, initial wiring costs are higher, primarily because the installation of the 15 pairs of wires at each station connector is tedious. Interface costs are intermediate. Current broadband cable access interfaces are more expensive than DISNET, but some ETHERNET interfaces have been announced which are comparable in cost. DISNET may be extended to longer distances with repeater amplifiers and can be designed to include branches with an appropriate 'T' circuit, but such enhancements are more complex than with serial coaxial cable techniques. A final disadvantage is that DISNET does not permit messages to be simultaneously broadcast to all stations, and therefore would require a system directory station if dynamic allocation of resources or electronic mail is needed.

### References

1. Kahn, R. E., *Proceedings of the IEEE*, **60** (1972), 1397.
2. Tanenbaum, A. S., *Computer Networks* (Prentice-Hall, Inc., New Jersey, 1981).
3. Abramson, N., *AFIPS Conference Proceedings*, **37** (1970), 281.
4. Metcalfe, R. M. and Boggs, D. R., *Communications of the Association for Computing Machinery*, **19** (1976), 395.

### LABORATORY EXHIBITION

The British Laboratory Ware Association, one of the principal trade associations covering UK suppliers of laboratory wares and equipment, has added its support to the London Laboratory Exhibition. The other sponsors are the Royal Society of Chemistry, the Scientific Instrument Manufacturers' Association, and the Chromatography Discussion Group. The exhibition's organizers are looking to expand internationally and are currently in discussion with European trade associations and professional bodies. 1984's show will be held in the Barbican, London, from 4 to 6 September (coinciding with Analyticon 84).

*More information from Curtis/Steadman and Partners Ltd, The Hub, Emson Close, Saffron Walden, Essex CB10 1HL, UK. Tel.: 0799 26699.*

### ANALYTICAL DIVISION, ROYAL SOCIETY OF CHEMISTRY

A meeting of the Analytical Division will be held at the University of Exeter on Thursday and Friday, 18 and 19 April 1985, to celebrate the long and distinguished career in analytical chemistry of *Professor E. Bishop.* Anyone wishing to take part in this meeting by submitting a paper is invited to communicate with *Miss P. E. Hutchinson, Analytical Division, Royal Society of Chemistry, Burlington House, London W1V 0BN.*

### STATISTICS IN ANALYTICAL CHEMISTRY

This is a meeting of the North West Region of the Analytical Division of the Royal Society of Chemistry and will be held on 14 March 1984 at the University of Lancaster, UK. Papers include:

Introduction to statistical techniques, by Derrick Chamberlain (ICI Ltd, Organics Division, Blackley, Manchester, UK).

Errors and repeatability, by Dick Boddy (Statistics for Industry [UK] Ltd, Knaresborough, North Yorks, UK).

Sampling, by John Sykes (ICI Ltd, Organics Division, Blackley, Manchester, UK).

Calibration, by Roland Caulcutt (Statistics for Industry [UK] Ltd, Knaresborough, North Yorks, UK).

Strategies for the optimization of experiments, by Dr Trevor Lilley (BP Research Centre, Sunbury-on-Thames, UK).

Application of statistics in analytical quality control, by Michael J. Gardner (Water Research Centre, Medmenham, UK).

*Prospective participants should contact Miss P. E. Hutchinson, Analytical Division, Royal Society of Chemistry, Burlington House, London W1V 0BN.*

### ERRATA

Mr Brian Wybrow has sent us some corrections to his paper on 'A microcomputer-based injection system for investigating the influence of atmospheric pressure on chromatographic response in the analysis of gases', which was published in Vol. 5, No. 3, pp. 124–135. They are:

On p. 129, 'Control of injection technique': 'Outline', 2nd paragraph, 2nd line, for responses read *response*; same page, 'Sample introduction', 2nd paragraph, line 7, for P1 read $P_1$; same page, 'Using the injection system': 'Outline', 1st paragraph, line 10, for AGPURGE read *AGCPURGE*.

On p. 131, 'Use of a printer', 1st paragraph, line 11, an *a* should be inserted between within and 'BASIC' on line 12.

On p. 134, 'Conclusions', 2nd paragraph, line 5, for $<0{\cdot}2\%$ read $<0{\cdot}02\%$.

There are mistakes, also, in one of the diagrams (*figure* 5). These are that SW 12 should be joined to Pin 7 of RY 8; Pin 1 of RY 10 should be joined to the + 5 V line from Interface 2 (as for Pin 1 of the other four relays); the lower, casing lines of the relays RY 7 and RY8 have breaks in them at the bottom left—they shouldn't; and, in the resistor network RN 3, the 'centre-line' from the mid-point position between Pins 1 and 14, to the mid-point position between Pins 7 and 8 is wrongly 'broken' between the 7–8 resistors and the adjacent pair—there should be a continuous line down the centre joining the centre points of the resistors.

Finally, in *table* 2, a dagger should have appeared next to 20 under 'Degrees of freedom (N-2)'.

